# Nanozymes in Tumor Microenvironment Modulation for Cancer Therapy

**DOI:** 10.34133/bmr.0425

**Published:** 2026-10-07

**Authors:** Xinyang Zhang, Ziqi Wang, Yangguang Niu, Jingjing Tong, Ranfeng Wang, Yulin Zhu, Pei Pei, Jie Li, Yunan Gao, Xiaoran Deng, Jundong Zhou

**Affiliations:** ^1^ Department of Radiooncology, the First Affiliated Hospital of Anhui Medical University, Hefei, Anhui Province 230022, China.; ^2^ Jiangsu Province Key Laboratory of Anesthesiology and Brain Science, Xuzhou Medical University, Xuzhou 221004, China.; ^3^ Department of Radiation Oncology, Zhongshan Hospital, Fudan University, Shanghai 200032, China.; ^4^ Key Laboratory of Radiation Damage and Treatment of Jiangsu Provincial Universities and Colleges, Soochow University, Suzhou 215123, China.

## Abstract

The tumor microenvironment (TME) plays a central regulatory role in malignant tumor progression and therapeutic resistance, and its complexity and dynamic heterogeneity markedly limit the efficacy of current treatment strategies. Therefore, the development of novel functional materials capable of effectively remodeling the TME and alleviating immunosuppression and therapeutic tolerance has become an important research direction in cancer therapy. In recent years, nanozymes, as a class of nanomaterials with enzyme-mimicking catalytic activity, have shown broad potential in TME intervention and the enhancement of cancer therapy owing to their excellent physicochemical stability, tunable structural properties, and multiple biomimetic enzyme-like activities. This review systematically summarizes recent advances in nanozyme-mediated regulation of the TME. First, the basic concepts, catalytic properties, and functional classifications of nanozymes are introduced. Subsequently, the mechanisms by which nanozymes remodel the TME and enhance cancer therapy are discussed from the key dimensions of the redox microenvironment, metabolic microenvironment, and immune microenvironment. Finally, the major challenges, barriers to clinical translation, and future development directions in this field are highlighted. This review aims to provide a theoretical basis and research perspectives for the further study, rational design, and translational application of nanozymes in cancer therapy.

## Introduction

The tumor microenvironment (TME) is a highly complex and dynamically evolving local ecosystem that emerges during tumor initiation, progression, and therapeutic adaptation. It is composed of tumor cells, immune cells, stromal cells, abnormal vascular structures, extracellular matrix (ECM), and diverse soluble factors [1,2]. A functional niche that supports tumor growth, invasion, metastasis, and resistance to therapy is established through multilayered signaling networks and material exchange among these components [3].

The TME contains a heterogeneous set of cell populations that remain in continuous communication [4], with malignant cells accompanied by tumor-associated macrophages, myeloid-derived suppressor cells, cancer-associated fibroblasts, and endothelial cells [5]. Cytokines, chemokines, and growth factors mediate much of this communication, sustaining tumor cell proliferation and survival. Immunosuppressive programs are reinforced at the same time, weakening antitumor immunity [6–8]. Vascular abnormalities compound these effects: Tumor vessels are poorly organized and excessively permeable, and inefficient perfusion leads to local hypoxia and the retention of metabolic waste [9–12]. These defects favor tumor progression while limiting the delivery and intratumoral distribution of therapeutic agents, including nanomedicines [13]. Metabolites and signaling mediators also shape this setting, with lactate and reactive oxygen species (ROS) helping tumors adapt to local stress and resist therapy. Their activity contributes to ECM remodeling and local acidification, while immune cell function is suppressed [14,15]. Explaining treatment failure at a biological level therefore requires these processes to be considered together; the same understanding can guide the design of more effective therapies [16].

These relationships have widened the therapeutic focus, bringing the microenvironment that sustains malignant cells into cancer treatment strategies [17]. Tumor cell elimination remains a central objective and is increasingly pursued alongside TME remodeling and activation of antitumor immunity [18]. Among these, TME-directed interventions, such as anti-angiogenic therapy and immunomodulatory approaches, have emerged as important means of overcoming therapeutic resistance and improving treatment efficacy [19]. As the heterogeneity of the TME has become more clearly appreciated, there is growing interest in precision and combinatorial therapeutic strategies tailored to specific microenvironmental features [20]. Therefore, achieving precise modulation of TME has been regarded as a key direction for improving cancer treatment outcomes [21].

The core biochemical characteristics of TME determine that it essentially constitutes a highly specific site of pathological biochemical reactions, and is generally characterized by biochemical imbalances such as overexpression of ROS such as hydrogen peroxide (H_2_O_2_), severe hypoxia, microenvironmental acidosis, high concentration of glutathione (GSH) barrier, and lactic acid accumulation [22–24]. Although these physicochemical and metabolic abnormalities are the causes of tumor progression and treatment resistance, from the perspective of catalytic medicine, they provide natural reaction substrates and microenvironment activation clues for catalytic intervention [25]. By transforming the pathochemical barrier of TME into an endogenous catalytic force in situ through an enzyme-like catalytic reaction, a novel intervention paradigm with endogenous response, high efficiency, and low toxicity will be provided for targeted remodeling of TME [26].

However, relying on natural enzymes to drive the abovementioned in situ catalytic processes is severely limited by the protein’s inherent inactivation, short in vivo half-life, and high production costs [27,28]. Therefore, “nanozymes”, which combine the stability of inorganic nanomaterials with intrinsic biomimetic catalytic activity, have emerged [29,30]. Compared with natural enzymes, nanozymes not only overcome the stability bottleneck under physiological conditions but also have important advantages such as strong structural controllability, low preparation cost, and ease of multifunctional integration [31–33]. Currently, nanozyme material systems mainly include metal oxides, noble metals, carbon-based materials, and metal–organic framework (MOF)-derived systems, which can efficiently mimic the activities of various natural enzymes such as peroxidase (POD), oxidase (OXD), catalase (CAT), and superoxide dismutase (SOD) [34,35].

With the aforementioned biomimetic catalytic properties and multifunctional integration capabilities, nanozymes can systematically reshape the complex TME network from core dimensions such as redox balance, metabolic programs, and immune microenvironment [31,36–39]. Therefore, current research is increasingly focused on the development of multimodal synergistic treatment systems, aiming to deeply integrate existing nanozyme catalytic therapy methods to achieve complementary advantages and synergistic enhancement of antitumor effects, and ultimately break through the limitations of single treatment modalities [40].

Nanozymes have shown considerable promise, although several practical issues continue to impede their clinical translation. Comprehensive in vivo biosafety assessment is required, and targeted delivery must be made more efficient. Catalytic selectivity also needs refinement, with performance established across heterogeneous tumor contexts [41,42]. Broader clinical use will further depend on scalable manufacturing and the establishment of appropriate regulatory standards [43].

Material classes and individual technologies provide the usual organizing structure for reviews in this field. Here, nanozyme-mediated remodeling of the TME is considered as an interconnected process. Interdependence among TME barriers guides the discussion, allowing the molecular routes through which pathological vulnerabilities become targets for catalytic intervention to be traced. This progression is framed in terms of biological dependence and ecological expansion. Redox regulation is considered first because it sets the biochemical conditions for the metabolic reprogramming discussed next. Immunological remodeling is then examined against this combined background, while vascular and microbial barriers extend the analysis to tissue structure and microbial ecology. The closing discussion assesses unresolved challenges and identifies priorities for future research.

The review is organized into 5 thematic parts: (a) regulation of the redox microenvironment; (b) regulation of the metabolic microenvironment; (c) regulation of the immune microenvironment; (d) regulation of other microenvironmental factors; and (e) summary and outlook (Fig. 1).

**Fig. 1.**
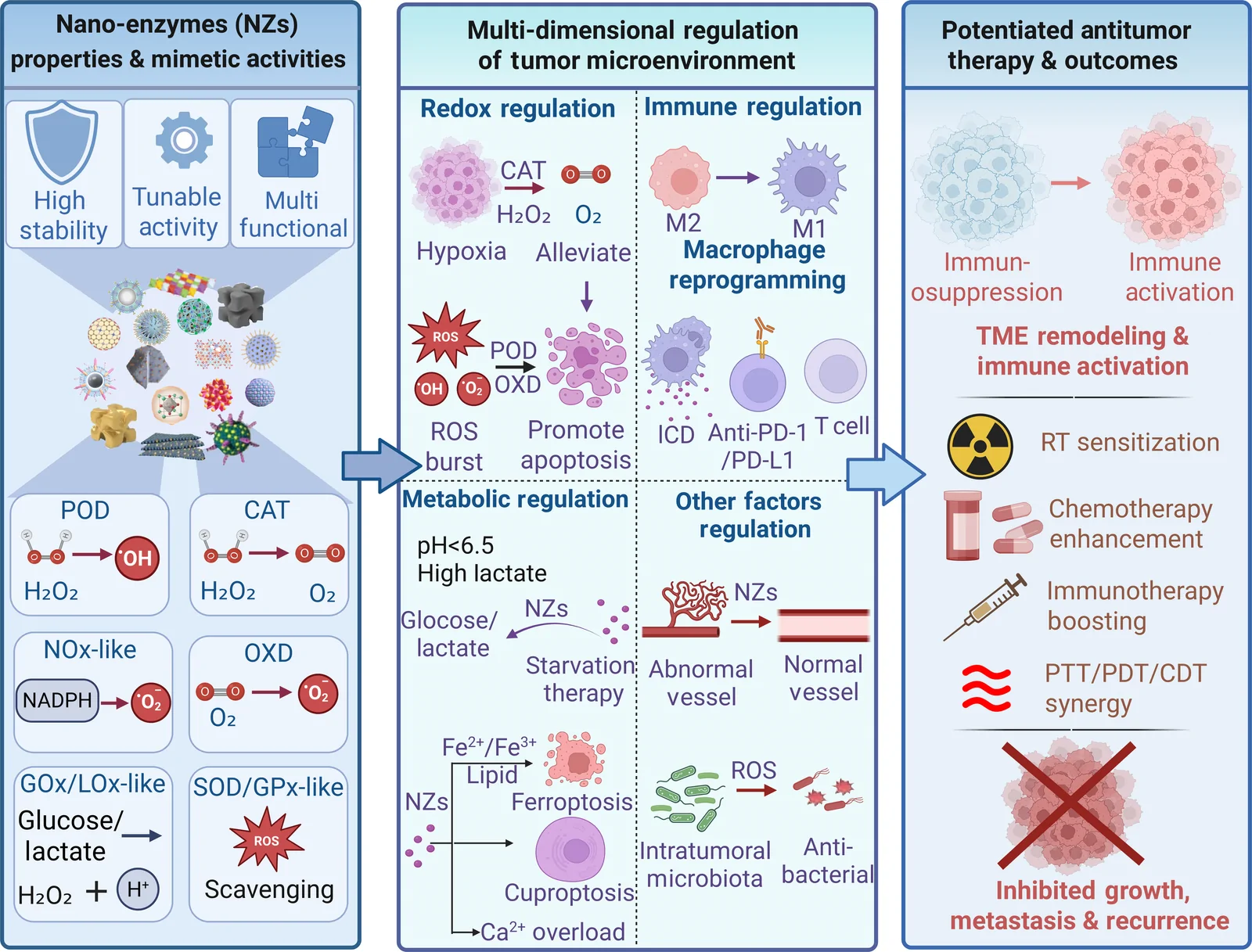
Nanozymes enhance cancer therapy through multidimensional modulation of the TME. Nanozymes remodel the TME by regulating redox balance, metabolism, immunity, angiogenesis, and microbiota, thereby enhancing therapeutic efficacy and supporting combination treatment strategies.

## Functional Classification of Nanozymes for Cancer Therapy

Nanozymes are functional nanomaterials whose active-site features and catalytic behavior resemble those of natural enzymes, enabling specific biochemical reactions to be catalyzed efficiently under physiological conditions. Leveraging their biomimetic enzymatic functions, nanozymes can be primarily classified into several categories within the field of tumor therapy:

a. ROS generating nanozymes: These include nanozymes that mimic the activities of POD, OXD, and NADPH (reduced form of nicotinamide adenine dinucleotide phosphate) oxidase (NOX) [44–48]. They can catalyze the generation of hydroxyl radicals from hydrogen peroxide [49,50], or they can directly activate oxygen to convert into superoxide anions and other ROS [51], thereby inducing severe oxidative stress within the TME and directly killing tumor cells [52]. Representative systems include CeO_2_-Mn_1.08_O*_X_* nanoclusters [53] and MoCu diatomic nanozymes [54].

b. Hypoxia-relieving nanozymes: These primarily mimic the activity of CAT [55,56], catalyzing the decomposition of overexpressed hydrogen peroxide within tumors to produce oxygen. This effectively alleviates tumor hypoxia, reverses hypoxia-related treatment resistance, and enhances the efficacy of radiotherapy as well as certain chemotherapies [57–59]. Examples include platinum–gold composite nanozymes and BiPt–folic acid-modified amphiphilic polyethylene glycol (BiPt-PFA) nanocomposites [60–62].

c. Metabolic intervention nanozymes: Glucose oxidase (GOx)-mimicking nanozymes use intratumoral glucose and molecular oxygen as substrates to catalyze the production of gluconic acid and hydrogen peroxide [63,64]. This catalytic reaction not only enables starvation therapy (ST) via energy depletion [65], but the co-produced gluconic acid also aggravates TME acidosis, while the generated hydrogen peroxide serves as a crucial cascade substrate to fuel a downstream ROS burst [66,67] (examples include Au/Ti/Fe-MOF composite hydrogels and copper single-atom nanozymes). Lactate oxidase (LOx)-mimicking nanozymes utilize the abundantly accumulated lactate and local oxygen in the TME as substrates to efficiently catalyze the generation of pyruvate and hydrogen peroxide. This biochemical transformation not only directly depletes lactate to mitigate microenvironmental acidosis and dismantle the immunosuppressive barrier, but the generated hydrogen peroxide also serves as a precursor fuel for subsequent catalytic therapies (examples include M/GLB@AuSAN and LOX-Ce6-Mn) [68,69]. Furthermore, glutathione peroxidase (GPx)-mimicking nanozymes mainly use antioxidant reduced glutathione and endogenous peroxides as substrates to catalyze their conversion into inactive oxidized glutathione (GSSG) and water [70]. This reaction, by drastically depleting the antioxidant buffer of TME, completely dismantles the oxidative stress defense network of tumor cells, thereby significantly enhancing their sensitivity to oxidative damage and ferroptosis [71,72] (examples include MET-CMS@FeTA and Ga/Zn diatomic nanozymes) [73].

d. Immunomodulatory and cascade-killing nanozymes: These nanozymes primarily mimic SOD and myeloperoxidase (MPO) [74]. Among these, MPO-like activity specifically utilizes endogenous hydrogen peroxide and chloride ions as co-substrates to catalyze the efficient generation of hypochlorous acid, a potent oxidant exhibiting pronounced cytotoxicity. By integrating the SOD-MPO catalytic cascade, this class of nanozymes faithfully recapitulates the neutrophil-mediated biochemical oxidative burst. This process triggers severe localized oxidative stress, directly inflicting irreversible DNA damage and driving apoptosis in tumor cells [75] (examples include Au_1_Pd_3_ sub-nanometer alloy nanozymes [76]).

The hypoxic, acidic, and hydrogen peroxide-concentrated characteristics of TME provide specific substrates and activation conditions for the catalytic reactions of the aforementioned nanozymes [77,78]. Nanozymes can thus act as “intelligent biocatalysts” within the TME, converting endogenous substrates in situ into highly reactive species. Specifically, they drive cascade reactions to generate ROS, thereby implementing robust chemodynamic therapy (CDT) [79], or improving hypoxia to sensitize radiotherapy and immunotherapy [60]. More importantly, through rational design and functional integration, a single nanozyme can simultaneously possess multiple enzyme activities and other therapeutic functions [80], thereby achieving multidimensional, programmable, and on-demand modulation of the TME [81]. This characteristic makes it promising to overcome the bottlenecks of traditional therapies such as strong drug resistance and poor selectivity, becoming a highly promising frontier direction in the field of precision oncology (Fig. S1).

Current research indicates that nanozymes regulate the TME primarily in 3 dimensions: the tumor redox microenvironment, the tumor metabolic microenvironment, and the tumor immune microenvironment. Based on this, nanozymes not only possess tumor treatment potential themselves but also can serve as an enhancement platform to synergize with various traditional and emerging treatment methods [82], including radiotherapy, chemotherapy, immunotherapy, photothermal therapy (PTT), CDT, photodynamic therapy (PDT), sonodynamic therapy (SDT) [83], ST, cryotherapy [84], boron neutron capture therapy (BNCT) [85–87], magnetothermal therapy (MHT) [88], and thermoelectric therapy [89]. In addition, the SOD-like activity of nanozymes can protect normal tissues from oxidative damage during treatment [90] and indicates potential advantages in inhibiting tumor recurrence and postoperative infection [91], demonstrating its significant value as a multifunctional treatment platform (Table 1).

**Table 1.** Nanozymes for TME remodeling and cancer treatment

Microenvironment	Enzyme activity	Nanozyme name	Mechanism of action	Treatment methods	Tumor models	Ref.
Tumor oxidation–reduction microenvironment	POD + CAT + GOx	BZAGTA	Decomposition of glycolysis-derived H_2_O_2_ generates O_2_ and ROS, relieving tumor hypoxia.	CDT + PTT + ST	4T1	[217]
	POD + CAT	MGNSs@Pt	Enhanced ROS and O_2_ generation required for near-infrared type II hyperthermia	CDT + PTT	4T1	[92]
	POD + CAT + GPx	CuPt	Catalytic H_2_O_2_ conversion and GSH consumption	Chemotherapy	4T1/A549R	[80]
	POD + CAT	UAPiGCH	Hypoxia relief by O_2_, tumor damage by ROS, synergy in CT and PTT	Chemotherapy + PTT	4T1	[77]
	POD + CAT + GPx	CeO_2_-enriched CuO	Catalytic H_2_O_2_ conversion and ROS generation, promotion of CT, CDT and oxidative stress	Chemotherapy + CDT	4T1	[78]
	POD + CAT + GOx	MnO2@PtAuRu	Glucose-mediated H_2_O_2_ generation, catalytic •OH production, GSH depletion, and CDT enhancement	CDT	HCT116	[49]
	POD + CAT	Au@Pt-Ce_6_-HN-1	Catalytic H_2_O_2_ conversion, synergistic promotion of PDT, PTT, and CDT	CDT + PDT + PTT	SCC9	[56]
	POD + OXD	PEG-b-PLL	SO_2_ release control and ROS accumulation	GT	4T1	[93]
	POD + GPx	Fe SAN-PDA@DOX@HA	Catalytic H_2_O_2_ conversion, ROS production, and GSH depletion	Chemotherapy + PTT	4T1	[82]
	POD + CAT + GOx	TANP	Glucose consumption, O_2_ generation from H_2_O_2_ decomposition, GSH depletion, ROS production	ST	4T1	[94]
	POD + CAT	FBFO@HM@aOPN	Catalytic H_2_O_2_ conversion, hypoxia relief, ROS storm, cuproptosis	Immunotherapy + PDT	CT2A	[115]
	POD + GPx + OXD	CFO-CUR	ROS production, Ca^2+^ accumulation, and induction of mitochondrial dysfunction	SDT	4T1	[157]
	POD + CAT	FZBNPs	O_2_ generation from H_2_O_2_ decomposition, LPO accumulation, GSH depletion, ferroptosis induction	PDT	4T1	[122]
	POD + GPx	Pt@MoS_2_	•OH generation via H_2_O_2_ and GSH decomposition, apoptosis induction, and ferroptosis induction	PTT	Huh7/MIHA	[134]
	CAT + OXD + NOX	Co-HCS/D/A	OXPHOS disruption, glycolysis blockade, and PPP inhibition via NADH oxidation	ST	HeLa	[160]
	POD + GPx	MIL-Cu_1.8_S-TPP/FA	Disruption of mitochondrial iron–sulfur proteins, cuproptosis induction, regulation of GPX4, GSH, FDX-1, and HSP70	PDT	4T1	[218]
	POD + SOD	1-NBS@CeO_2_	ROS production, GSH depletion, mitochondrial dysfunction, apoptosis and ferroptosis induction	PDT	A375	[132]
	NOX	BV	Hypoxia-induced NAD^+^/NADH imbalance, OXPHOS inhibition, glycolysis blockade	CDT + ST	4T1	[161]
	POD	DPHCO	ROS production, LPO accumulation, GSH depletion, GPX4 down-regulation, ferroptosis promotion	NCT	4T1	[135]
	ChOx	Cu-HPB/C	H_2_O_2_ generation from cholesterol consumption, cholesterol homeostasis disruption, lipid valve dysfunction	CDT	4T1	[162]
	POD + CAT + GPx + OXD	FeNC-FA	ROS storm induction, GSH depletion, ferroptosis initiation, HSP suppression, and mPTT enhancement	PTT	4T1	[138]
	POD + GPx + NOX	CuSAE	NADPH depletion, •OH production, GSH depletion, disulfide bond formation, apoptosis induction	ST	4T1	[127]
	CAT + GPx	Ru/TiO_2-x_ SAE	ROS generation, LPO accumulation, ferroptosis induction	SDT	GL261	[136]
	POD + OXD	GAFe@DOX	ROS generation via H_2_O_2_ decomposition, oxidative stress induction, and excessive autophagy, ferroptosis, and apoptosis	Chemotherapy	GL261/HUVECs	[137]
	POD + CAT + GOx	GPGP	Catalytic H_2_O_2_ conversion, •OH production-induced ferroptosis, and radiotherapy sensitization	RT	4T1	[121]
	POD + CAT + GOx	AuPt NPs@OXA gel	Glucose breakdown into gluconic acid and H_2_O_2_, followed by H_2_O_2_ decomposition into O_2_ and ROS	Chemotherapy + PTT + CDT + Immunotherapy	4T1	[60]
	POD	ZnO_2_ @Pt	ROS production, oxidative damage, apoptosis induction, glycolysis inhibition	Ion interference + CDT	4T1	[107]
Tumor immune microenvironment	CAT + SOD	I@P@HP-tLyp-1	Immune cell targeting, M1 macrophage polarization promotion, and Treg activation reduction	Immunotherapy	CT26	[219]
	CAT	BBOP	ROS production, PANoptosis/ICD induction, O_2_ generation from H_2_O_2_ decomposition, ultrasound therapy enhancement	Sonocatalytic immunotherapy	4T1	[109]
	GPx	Ag/Fe-HfO_2_-PEG-RGD	O_2_ and •OH production, GSH depletion, and up-regulation of tumor-infiltrating CD8^+^ T cells	RT + PTT	4T1	[72]
	POD + CAT	Mn^2+^@AuPdRh	cGAS–STING activation, O_2_ generation from H_2_O_2_, •OH production, ICD induction	Immunotherapy	B16F10	[119]
	POD	M@MnO_2_-Au-mSiO_2_@CDDP	ROS generation, STING activation	Immunotherapy	MFC	[184]
	POD + GPx + OXD	FCMP@CQ/PFH	Redox stress induction, lysosomal inhibition, autophagy suppression, and antitumor immune activation	Immunotherapy	MC38	[71]
	POD + CAT + GPx	Q-MIL-53(Fe)	Ferroptosis induction, ICD induction, DC maturation promotion, T cell infiltration enhancement	Immunotherapy	4T1	[176]
	POD + CAT + GPx + SOD	IMZF	H_2_O_2_ decomposition-mediated O_2_ generation, hypoxia alleviation, macrophage polarization, ROS generation, GSH depletion, ICD induction, DC activation, inflammatory lymphocyte infiltration	Immunotherapy	4T1/MCF-7	[167]
	POD + CAT + GPx	MnO_2_-x/HPB	GSH depletion, H_2_O_2_ decomposition-mediated O_2_ and ROS generation, hypoxia alleviation, ICD induction, cGAS–STING activation	SDT + CDT	4T1	[79]
	POD + CAT	MNPT	ROS production, GSH modulation, T cell exhaustion, apoptosis induction, immune response activation	PDT + Immunotherapy	4T1	[197]
	POD + CAT + GPx	FeCu-DA	O_2_, •OH, and H_2_O_2_ generation, GSH depletion, ICD induction	PTT + Immunotherapy	4T1	[182]
	POD + GPx + SOD	HE-LDH	ROS production catalysis, Zn^2+^ release, cGAS–STING activation, antitumor immune promotion	NCDT + Immunotherapy	4T1	[70]
	POD + GPx	RePd@OMVs^PD-L1 nb^	ROS clearance inhibition, oxidative stress enhancement, ICD and apoptosis induction, immune activation	PTT + Immunotherapy	MB49	[177]
	POD	Fe-MOF/CP	ROS production, LPO accumulation, ferroptosis induction, ICD induction, PD-L1 down-regulation, T cell exhaustion reversal	Immunotherapy	4T1	[178]
Other tumor microenvironments	POD + GOD + GPx	CeO_2_@GNSs/Myr-HA	VEGF expression inhibition, tumor angiogenesis suppression via p38/MAPK pathway	Chemotherapy + PTT	HeLa	[200]
	CAT + NOS	HRRu-HFn	Endogenous H_2_O_2_-triggered NO and O_2_ release, tumor angiogenesis improvement, oxygenation enhancement, radiosensitivity promotion	RT	EC109	[206]
	POD + GOx + SOD	FGA@Fu	ROS release, resting vascular endothelial cell activation	GT + PTT	4T1	[201]

## Nanozyme-Mediated Modulation of the Tumor Redox Microenvironment

The tumor redox microenvironment is defined as the dynamic local environment with unique biochemical characteristics inside tumor tissue. Its core manifestation is a continuous increase in ROS levels, while the antioxidant system is abnormally activated. Together, the 2 maintain a fragile and readily disrupted redox balance. The formation of this microenvironment is closely related to the metabolic reprogramming of tumor cells and the interaction with the surrounding matrix. Therapeutic disruption of this unstable balance can induce tumor cell death and may therefore have translational value [92]. Nanozymes are suited to this task because their catalytic activity can be tuned and multiple functions can be integrated into a stable platform. These properties allow the tumor redox state to be regulated efficiently and with greater precision. Tumor cells typically withstand elevated ROS levels by strengthening their antioxidant defenses. Nanozymes can be engineered to accumulate preferentially at tumor sites, where redox homeostasis is disrupted through several catalytic routes [93]. Their tunable catalysis and biocompatibility have supported their development as platforms for regulating the tumor redox microenvironment [82] (Fig. 2).

**Fig. 2.**
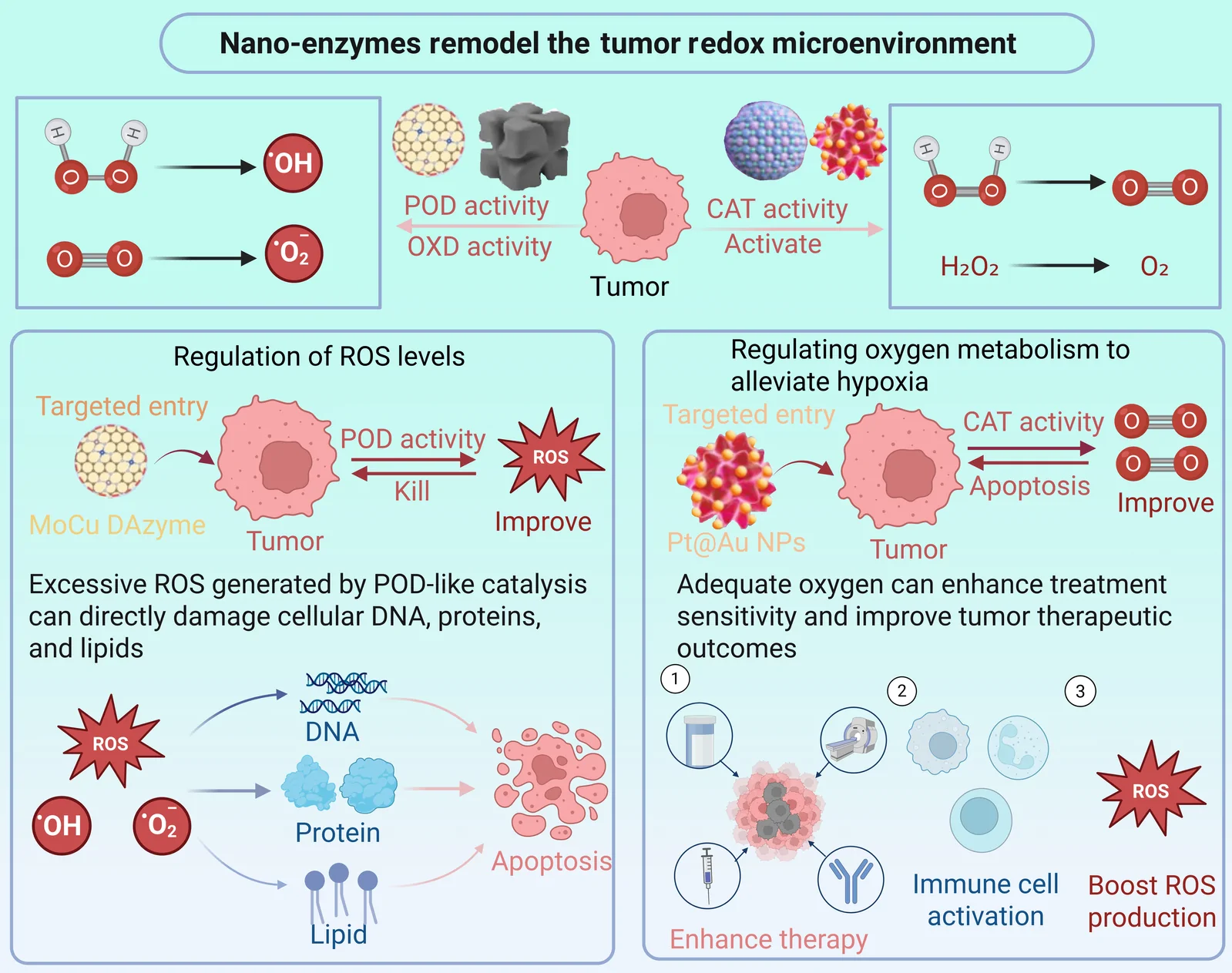
Nanozyme-mediated modulation of the tumor redox microenvironment. Nanozymes regulate the tumor redox microenvironment through 2 major routes: ROS generation to induce oxidative damage in tumor cells, and oxygen generation to relieve hypoxia and enhance ROS-dependent therapies. Through their combined action, redox homeostasis is disrupted, thereby increasing therapeutic responsiveness.

### Regulation of ROS homeostasis

ROS-generating nanozymes can profoundly dismantle tumor redox homeostasis via 4 distinct catalytic mechanisms. Specifically, (a) POD-like activity drives the conversion of hydrogen peroxide into highly toxic hydroxyl radicals [94,95]; (b) OXD-like activity mediates the single-electron reduction of molecular oxygen into superoxide anions [96]; (c) NADPH oxidase (NOx)-like pathways further contribute to superoxide anion generation [54,97]; and (d) GOx-like properties synergistically exacerbate the intratumoral oxidative stress [98]. Through the above synergistic catalysis, nanozymes introduce a massive amount of exogenous ROS into the tumor site. The resulting ROS accumulation can exceed the cellular tolerance threshold, causing severe oxidative damage and tumor cell death through apoptosis, necrosis, or related pathways [99,100].

Design strategies for ROS-modulating nanozymes have evolved as the physicochemical basis of catalysis has become clearer. Early studies relied largely on empirical material screening and individual catalytic modes. Current designs often place several biomimetic active sites in the same platform, reducing constraints imposed by substrate availability and allowing separate catalytic centers to operate in cascade. Qiao et al. [53] used this arrangement to construct CeO_2_-Mn_1.08_O*_x_* nanoclusters with POD- and OXD-like activities. Li and coworkers [54] incorporated POD-, OXD-, NOX-, and GPx-like activities into a Mo–Cu diatomic nanozyme. ROS production was amplified, while GSH regeneration was suppressed, together disturbing tumor redox balance through complementary catalytic routes. Reaction kinetics can also be altered by adjusting the electronic structure of active sites at the atomic scale. Adsorption and activation of reaction intermediates are changed in the process, directly affecting catalytic turnover. By optimizing the d-band center of Fe_1_Mn_1_-NCe diatomic sites, Zeng and colleagues [101] improved catalytic performance while increasing structural stability. Surface morphology and stereochemistry provide additional control over substrate recognition. Kang et al. [102] reproduced the stereoselective recognition of natural enzymes using D-configured gold nanoparticles irradiated with circularly polarized light. A 2-fold increase in substrate-binding selectivity was achieved. Across these studies, rational structural engineering was linked to gains in catalytic selectivity and efficiency [103–107].

### Modulation of tumor oxygenation and hypoxia relief

Hypoxia-relieving nanozymes can catalyze the decomposition of hydrogen peroxide to generate oxygen in the TME through their CAT-like activity, thereby significantly increasing local oxygen levels and improving the hypoxic state [108]. This process not only helps to alleviate hypoxia-mediated treatment resistance but also promotes the infiltration and activation of immune cells and enhances the antitumor immune response [50,109]. It is worth noting that oxygen is an essential substrate for the generation of ROS, and hypoxia will severely limit the efficiency of ROS generation. Therefore, nanozymes can increase the oxygen content in the TME through CAT activity [60], directly alleviating hypoxia and providing a basis for the subsequent large-scale generation of ROS, which further enhances tumor cell killing by elevating oxidative stress levels [47,110,111].

To address the mass transfer and kinetic bottlenecks caused by deep hypoxia and low conversion of endogenous H_2_O_2_ in the TME, the design strategy for improving the efficacy of hypoxia-relieving nanozymes has also undergone significant innovation.

Engineering heterogeneous interfaces represents an effective way to improve catalytic performance by promoting intercomponent interactions. The charge redistribution and accelerated electron transfer across heterogeneous interfaces can regulate substrate adsorption and activation, thereby reducing the kinetic barriers involved in catalytic reactions. Enhanced O_2_ generation was observed for the Pt@Au bimetallic heterostructure [61] and the 2-dimensional (2D) Ti_3_C_2_T_x_-AuX-PEG composite [94]. Improved interfacial charge transfer and cooperation between catalytic components were considered responsible for this behavior. Asymmetric spatial design provides a separate way to relieve mass transport limitations in the TME. Gas produced during catalysis may collect near exposed active sites, where it blocks access and interrupts the continuous supply of substrates. The Au2Pt@MnO_2_@ICG Janus nanomotor developed by Chen and coworkers [112] uses asymmetric gas generation as a propulsion mechanism. This motion promotes intratumoral penetration and improves substrate transport within tumor tissue.

When substrates are scarce, defect engineering may be paired with an external energy input to sustain catalytic reactions. Lattice defects alter the electronic structure of catalytic centers, while the external stimulus supplies the energy required to overcome high activation barriers. Li et al. [113] integrated oxygen-vacancy-rich ZnSnO*_x_*:Mn with ultrasound-driven piezocatalysis, enabling oxygen to be produced from water splitting through an alternative catalytic pathway. In the BiPt-PFA composite, local temperature is raised through photothermal conversion; the accompanying increase in molecular mobility accelerates catalytic kinetics [62]. In addition, the Lu-Au–Pt@PCNs (APPs)-PEG platform developed by our group combines radioisotope-mediated irradiation with oxygen-regulating components, allowing in situ modulation of the TME and alleviation of hypoxia-associated radioresistance [114]. Collectively, these advances highlight the importance of rational structural design and external-field regulation in developing oxygen-modulating catalytic systems for tumor therapy [115–118].

Nanozymes can precisely regulate the tumor redox microenvironment and utilize their ROS to achieve targeted therapy [105]. Multi-enzyme synergistic catalysis contributes to overcoming the limitations of traditional therapies, demonstrating significant potential in reversing drug resistance and enhancing immunotherapy [119,120].

Despite significant progress, the field still faces many serious challenges. First, the absolute concentration of redox-active substrates within the TME is intrinsically low and exhibits pronounced spatiotemporal heterogeneity, which severely hampers substrate-dependent cascade catalysis, particularly in deep-seated hypoxic/anoxic regions. Secondly, most nanozymes currently have lower substrate specificity than natural enzymes and are prone to nonspecific oxidative stress in complex body fluid environments. Concurrently, tumor cells dynamically up-regulate their endogenous antioxidant defense network to elicit compensatory adaptations, thereby effectively scavenging exogenous ROS. Therefore, future mechanistic exploration should focus on developing orthogonal catalytic nanozyme systems with substrate self-sufficiency or the ability to dynamically respond to extreme fluctuations in the microenvironment, thereby overcoming the limitations of local redox buffer barriers.

The intervention of nanozymes on tumor redox homeostasis is not an isolated biological event, but is closely coupled with tumor metabolic reprogramming. Dramatic fluctuations in redox state can directly reshape the metabolic flux of tumor cells: On the one hand, the oxygen supply brought about by CAT-like activity can alleviate hypoxia and promote the redistribution of tumor energy metabolism flux; on the other hand, the dramatic accumulation of ROS can damage the mitochondrial respiratory chain and induce lipid peroxidation, providing the necessary substrates and conditions for the regulation of ferroptosis and other ion homeostasis. Therefore, in-depth exploration of the metabolic cascade effects after nanozymes remodel the redox microenvironment is an inevitable extension for achieving multidimensional synergistic intervention in the TME.

## Nanozyme-Mediated Remodeling of the Tumor Metabolic Microenvironment

The tumor metabolic microenvironment is a dynamic and complex system characterized by hypoxia. It relies on aerobic glycolysis and glutamine as the main energy and carbon source supply pathways, and meets biomass requirements through de novo synthesis of lipids and amino acids. At the same time, it depends on metal ions such as iron and copper as cofactors for metabolic enzymes. Tumor cells acidify the local milieu by exporting lactate and protons. Essential amino acids are consumed continuously as well, and the reduced availability of arginine and tryptophan limits their use by immune cells and compromises immune function. Metabolic reprogramming depletes nutrients and allows waste products to accumulate, with extracellular acidosis developing in parallel. The altered environment favors immune escape and resistance to therapy. Within the resulting niche, tumor survival, invasion, and metastasis are supported, while normal cellular activity is progressively suppressed. Nanozymes intended for metabolic intervention have been investigated as tools for remodeling this environment. Multiple catalytic activities can be programmed within these systems, and their regulatory functions can be directed toward defined targets. By altering oxygen availability and the metabolism of glucose or lactate [121], and by regulating iron and copper homeostasis [122,123], they may overcome metabolic barriers that constrain conventional therapy (Fig. 3).

**Fig. 3.**
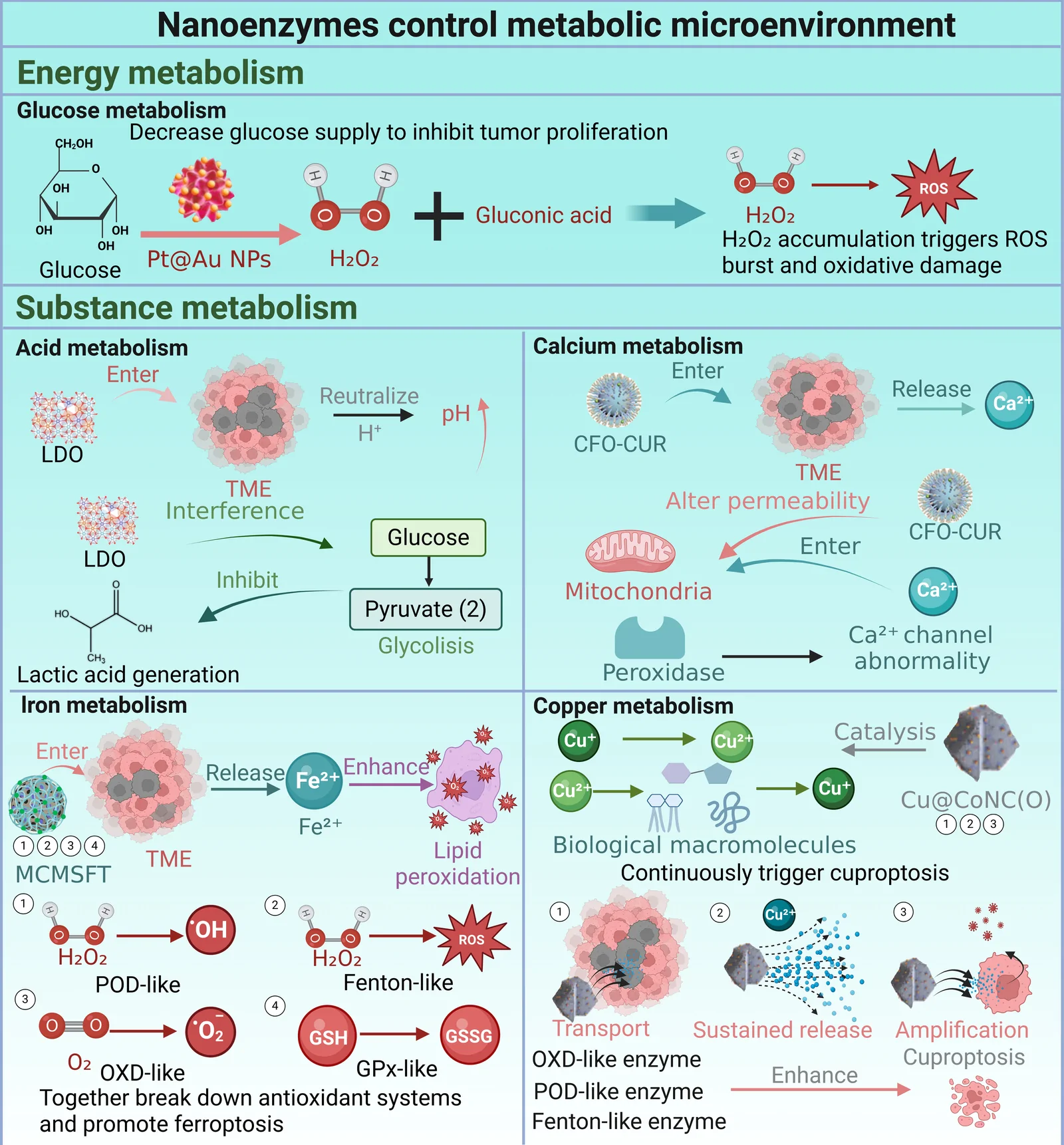
Nanozyme-mediated remodeling of the tumor metabolic microenvironment. Nanozymes modulate tumor metabolic reprogramming by regulating energy and lactate metabolism together with the homeostasis of iron, copper, and calcium. Substrate depletion and metabolic disruption are coupled with the induction of regulated cell death, thereby reshaping the metabolic microenvironment and suppressing tumor progression.

### Regulation of energy metabolism

Within the TME, aerobic glycolysis is favored by tumor cells even under oxygen-rich conditions, a metabolic phenotype known as the Warburg effect. During this process, glucose is rapidly converted to lactate, which then accumulates locally. Rapid glucose consumption restricts its availability to immune cells, impairing their metabolism and weakening antitumor activity. Lactate-driven acidosis further reinforces immunosuppression and supports tumor progression. Metabolic intervention nanozymes can catalyze the oxidation of glucose to gluconic acid through GOx-like activity, consume local glucose, implement “starvation therapy”, and inhibit tumor energy acquisition and biosynthesis [124,125]; at the same time, lactic acid production is reduced, acidosis is relieved, and immune cell function is restored [69], thereby enhancing the overall therapeutic effect while inhibiting tumor progression [126].

Recently, nanozymes have emerged as a powerful platform for the precise modulation of glucose metabolism. The self-cascaded Pt@Au nanozyme developed by Zhang’s group [61] has both GOx-like and CAT-like activities, catalyzing oxygen production while consuming glucose, thus enhancing the effect of ST. The biomimetic single-atom nanozyme system constructed by Zhang et al. [84] can activate brown adipose tissue to competitively take up glucose, deepening tumor metabolic starvation; the Au/Ti/Fe-MOF composite hydrogel system, developed by Xu et al. [67], integrates a quadruple synergistic therapeutic modality that encompasses SDT, CDT, ST, and chemotherapy.

Furthermore, nanozymes can modulate glucose metabolism to trigger disulfidptosis. In the synergistic strategy developed by Yu and coworkers, which integrates copper single-atom nanozymes (CuSAE) with GOx, a dual-pronged approach is employed to trigger disulfidptosis. Specifically, GOx induces glucose starvation, thereby impairing NADPH regeneration via the pentose phosphate pathway. Simultaneously, CuSAE functions as a NOX mimic to actively deplete the remaining NADPH pool. Simultaneous inhibition of these 2 components of the disulfide reduction system promotes disulfidptosis in tumor cells [127].

Preferential glucose utilization by tumor cells can also be disrupted, relieving competition for metabolic resources in the TME and making more nutrients available to infiltrating immune cells, whose antitumor activity is thereby better sustained. Nanozyme-mediated treatment therefore combines tumor nutrient deprivation with regulation of metabolic exchange across multicellular systems, providing a rationale for its use in combination therapy [128].

### Regulation of metabolite and ion homeostasis

#### Lactate metabolism

Lactate oxidation is also catalyzed directly through the intrinsic LOx-like activity of these nanozymes, complementing their indirect modulation of glucose metabolism. This process converts lactate and oxygen into pyruvate and hydrogen peroxide, thereby effectively alleviating lactate accumulation. Some nanozyme materials or their surface functional groups can function as proton scavengers to neutralize local hydrogen ions, elevate the pH value of TME, and reverse the immunosuppression and treatment resistance caused by acidosis [129]. The above strategies further enhance the synergistic therapeutic effects of nanozymes.

Over the past few years, significant strides have been made in leveraging nanozymes to reprogram lactate metabolic pathways and regulate the acidity of the TME. The ComnFe layered double oxide nanosheets designed by Jia’s group [130] have multi-enzyme activity, which can effectively deplete lactate and inhibit the repair of tumor cell DNA and protein. Meanwhile, the clearance of lactate and the deacidification of the microenvironment directly relieve the metabolic inhibition of neighboring immune cells and promote the reprogramming of tumor-associated macrophages (TAMs) to the antitumor M1 type, thereby synergistically enhancing the radiotherapy effect [24]. In addition to directly exerting LOx-like activity, nanozymes also show promise as carriers for LOx. Wang and coworkers assembled the M/GLB@AuSAN biomimetic composite by combining gold-based single-atom nanozymes with GOx and LOx. Under aerobic conditions, the system efficiently oxidizes lactate, and hydrogen peroxide is formed as a by-product. A separate platform reported by Wang et al. [68], LOX-Ce6-Mn, converts endogenous H_2_O_2_ into O_2_ through CAT-like catalysis, thereby sustaining lactate depletion. Liang’s team [131] targeted acidity using the CaCO_3_@Pd@C biomineralization system; as hydrogen ions are consumed, local pH rises and acidity in the metabolic microenvironment is relieved. These systems act at different points in tumor metabolism and may inform the design of combination therapies.

#### Iron homeostasis and ferroptosis

The elevated iron uptake and accumulation characteristic of tumor cells provide a basis for nanozyme-mediated regulation of iron metabolism. As the intracellular iron pool expands, Fenton conversion of hydrogen peroxide into hydroxyl radicals is accelerated. Lipid peroxidation follows and eventually induces ferroptosis. In terms of specific strategies, nanozymes serve as efficient iron carriers for the direct delivery of exogenous iron ions. Furthermore, through their GOx- and POD-like activities, these platforms continuously supply the essential substrates required for the Fenton reaction, thereby significantly accelerating the generation of hydroxyl radicals [132,133]. In addition, some nanozymes have GPx-like activities, which can deplete reduced GSH, weaken the antioxidant defense of tumor cells, and relieve resistance to ferroptosis. The above mechanisms work together to construct a highly efficient ferroptosis-inducing microenvironment in the tumor, providing a new way to enhance the efficacy of tumor therapy [134–138].

In recent years, nanozymes have shown multi-mechanism synergistic potential in regulating iron metabolism and inducing ferroptosis. The multifunctional nanozyme MET-CMS@FeTA developed by Zhang’s team [73] achieves a synergistic effect of oxygen self-supply, hydroxyl radical generation, and GSH depletion through multi-enzyme activity, effectively alleviating tumor hypoxia and activating the ferroptosis pathway. He et al. [139] designed a dual-targeting nanozyme MH-iRGD (an iRGD-functionalized, modified hyaluronic-acid-coated manganese ferrite nanozyme) that can down-regulate key ferroptosis regulatory proteins such as SLC7A11 and GPX4 under near-infrared light irradiation, and regulating Bcl-2/Bax apoptosis-related proteins, thereby achieving synergistic activation of ferroptosis and apoptosis.

It is worth noting that the intense lipid peroxidation triggered by ferroptosis, while causing tumor cells to disintegrate, releases a large number of damage-associated molecular patterns (DAMPs), which in turn cross cell boundaries to activate dendritic cells (DCs) and initiate an adaptive immune response [140].

Besides iron, other nonferrous metals can also induce ferroptosis through the “pseudo-iron effect”. At the coordination chemistry level, trivalent gallium ions and high-spin trivalent iron ions are both hard acids, and they exhibit high similarity in ionic radius, octahedral coordination geometry, and ligand affinity. In the cascade catalysis process, Ga^3+^ can displace bound iron ions from binding sites such as ferritin through thermodynamic competition, leading to a rapid expansion of the labile iron pool within the cell. Subsequently, the surge in free iron further triggers a highly efficient Fenton reaction and accumulates lipid peroxides, ultimately activating the self-amplified ferroptosis pathway. The Ga/Zn diatomic nanozyme reported by Zhong et al. [141] not only mimics POD and GPx to achieve cascade catalysis, but the gallium ions it releases also activate the self-amplified ferroptosis pathway by interfering with cellular iron metabolism homeostasis, providing a new perspective for expanding the ferroptosis induction mechanism.

Rational nanozyme design allows ferroptosis to be controlled at several points in the pathway and may broaden its application in cancer therapy [142–145].

#### Copper homeostasis and cuproptosis

Metabolic intervention nanozymes induce cuproptosis by disturbing copper homeostasis. At the molecular level, excess Cu^2+^ in mitochondria binds directly to lipoylated proteins involved in the tricarboxylic acid cycle. Abnormal protein aggregation and proteotoxic stress follow, eventually causing metabolic dysfunction and cell death [146]. Nanozymes have also been investigated as carriers for copper ions, allowing Cu^2+^ to be transported to mitochondria and released at elevated local concentrations [147]. Their GPx-like activity provides an additional mechanism by depleting GSH and thereby limiting cellular copper sequestration. When labile copper rises beyond the intracellular buffering limit, the cuproptosis pathway is engaged [148–150]. Therapeutic efficacy may therefore be improved by combining ion delivery with catalytic regulation.

Pairing copper delivery with catalytic control may consequently strengthen therapeutic efficacy. Nanozyme platforms are increasingly designed to combine regulation of copper metabolism with ferroptosis-related mechanisms, bringing cuproptosis into combination regimens. Our group developed the copper-loaded oxygen generator ^Cu/AP^H-M, which improves tumor oxygenation and has enabled the therapeutic contribution of cuproptosis to be examined [117]. Liu’s team [151] took advantage of the elevated hydrogen sulfide level in colorectal tumors to construct a TME-responsive platform. This endogenous trigger initiates a catalytic cascade in which ROS-mediated apoptosis is coupled to cuproptosis caused by copper overload. Kim et al. [152] pursued a related metabolic strategy using an oxygen-enriched cobalt single-atom nanozyme. Its CAT-like activity relieves hypoxic stress and redirects metabolism from glycolysis to mitochondrial respiration, increasing tumor cell sensitivity to cuproptosis under oxygen-limited conditions.

Coordinated regulation of copper metabolism and ferroptosis can produce complementary therapeutic effects. The Fe^3+^/Cu^2+^ dual-ion-site single-atom nanozyme developed by Xu’s team [153] promotes ROS generation while depleting cellular antioxidants. Ferroptosis and cuproptosis are consequently induced together, accompanied by increased tumor immunogenicity. In addition to contributing to tumor cell elimination, this dual-metal intervention causes a cascade of metabolic disturbances, some of which may serve as endogenous immune signals. Immune cells with antitumor activity are then recruited and activated in response to the increase in tumor immunogenicity [154]. A bifunctional nanoheterojunction containing iron and copper was reported by Liu et al. [155], enabling coordinated activation of ferroptosis and cuproptosis. Zhang and coworkers [156] examined the interaction between these cell death pathways using a copper-doped hollow Prussian blue nanozyme. In this system, copper induces the aggregation of metabolic enzymes and disrupts iron–sulfur cluster proteins, thereby promoting cuproptosis. Elevated oxidative stress concurrently drives lipid peroxidation and suppresses GPX4, favoring ferroptosis. The 2 pathways reinforce one another and improve antitumor efficacy.

#### Calcium homeostasis and calcium overload-associated cell death

In tumor cells, calcium signaling pathways are aberrantly up-regulated to support malignant proliferation. When calcium homeostasis is severely disrupted, however, calcium shifts from a regulatory mediator to a cytotoxic factor and causes irreversible cellular damage. Based on this concept, degradable calcium-based nanomaterials, such as calcium phosphate nanozymes, have been developed to utilize their tumor-targeting accumulation and acid-responsive degradation properties for controlled calcium ion release, thereby inducing a “calcium storm” [157]. The excessive calcium influx subsequently causes mitochondrial calcium overload and triggers endoplasmic reticulum stress. Activation of calcium-dependent enzymatic pathways further aggravates membrane disruption and energy metabolism dysfunction, leading to calcium-dependent cell death [75,158].

The 2D Ca_2_Mn_8_O_16_ nanosheets developed by Wang and coworkers [159] enable exogenous Ca^2+^ release and ROS generation through POD- and OXD-like catalytic activities. The elevated ROS level further promotes endogenous Ca^2+^ accumulation, resulting in the coordinated induction of apoptosis and suppression of tumor metastasis. The zinc-based nanozyme composite system constructed by Zhang et al. [158] regulates calcium metabolism through ROS, binds to exogenous Ca^2+^, induces mitochondrial dysfunction, and inhibits tumor progression. The enzyme-driven nanozyme system developed by Chang’s team [157] enhances the antitumor effect by using curcumin to inhibit Ca^2+^ efflux, thereby increasing mitochondrial calcium accumulation. These studies provide new insights into antitumor strategies based on calcium signaling regulation.

Nanozymes, through their enzyme-mimetic catalytic activities, have been widely explored for regulating tumor metabolic reprogramming and show potential in metabolic interventions, including substrate depletion and disruption of ion homeostasis [160–162]. However, a major challenge in metabolic regulation strategies arises from the high metabolic plasticity and adaptive capacity of tumor cells. When glucose metabolism is inhibited as a single intervention, tumor cells may adapt rapidly and maintain their energy supply through increased glutamine utilization or lipid oxidation [163]. Similarly, therapeutic approaches targeting ferroptosis or cuproptosis can be attenuated by activated metal efflux systems and compensatory antioxidant mechanisms. In addition, the pronounced heterogeneity of the tumor metabolic microenvironment further complicates the outcome of metabolic interventions. Future studies should therefore combine single-cell metabolomic analysis with isotope-based metabolic flux tracing to better characterize dynamic metabolic adaptation networks. A deeper understanding of these processes may facilitate the development of catalytic platforms that simultaneously regulate dominant metabolic pathways and compensatory metabolic routes [107].

Remodeling the tumor metabolic microenvironment can restrict malignant cell growth while alleviating metabolic suppression in immune cells. Immunosuppressive metabolites and nutrient competition are major contributors to tumor immune escape; nanozymes can therefore be used to regulate local metabolic networks through the modulation of energy metabolism, lactate degradation, and metal ion homeostasis. Such metabolic regulation has been shown to facilitate DC maturation, promote macrophage polarization toward the M1 phenotype, and enhance the infiltration of cytotoxic T lymphocytes. Metabolic intervention therefore contributes to tumor growth control and provides an important foundation for reshaping the immune microenvironment through improved metabolic conditions and immune responsiveness.

## Nanozyme-Mediated Reprogramming of the Tumor Immune Microenvironment

The tumor immune microenvironment (TIME) contains a broad range of immune cells, among them T cells, natural killer (NK) cells, macrophages, and DCs. Cytokines and chemokines form its soluble compartment, while antibodies and the ECM contribute acellular elements. One of its defining properties is sustained immunosuppression, as tumor cells remodel adjacent immune populations and redirect their activity toward a tumor-supportive state. Through continuous interactions with malignant cells, the TIME contributes to tumor growth, invasion, and metastasis; responses to treatment are weakened at the same time. Two mechanisms are especially important: increased expression of checkpoint molecules such as PD-L1 and secretion of inhibitory cytokines such as transforming growth factor-β (TGF-β) and interleukin-10 (IL-10). Regulatory T cells and myeloid-derived suppressor cells are also recruited, while competition for metabolic resources adds another layer of immunosuppression. Because their catalytic behavior and tissue targeting can be tailored, nanozymes are being investigated as regulators of these immune processes, including the removal of immunosuppressive factors and enhancement of antigen presentation. Costimulatory signaling may also be modulated, allowing immune cell function and homeostasis to recover [164]. The combined effect may shift an immunosuppressive “cold” tumor toward an immune-active “hot” state [165], thereby countering immune escape and improving antitumor efficacy (Fig. 4).

**Fig. 4.**
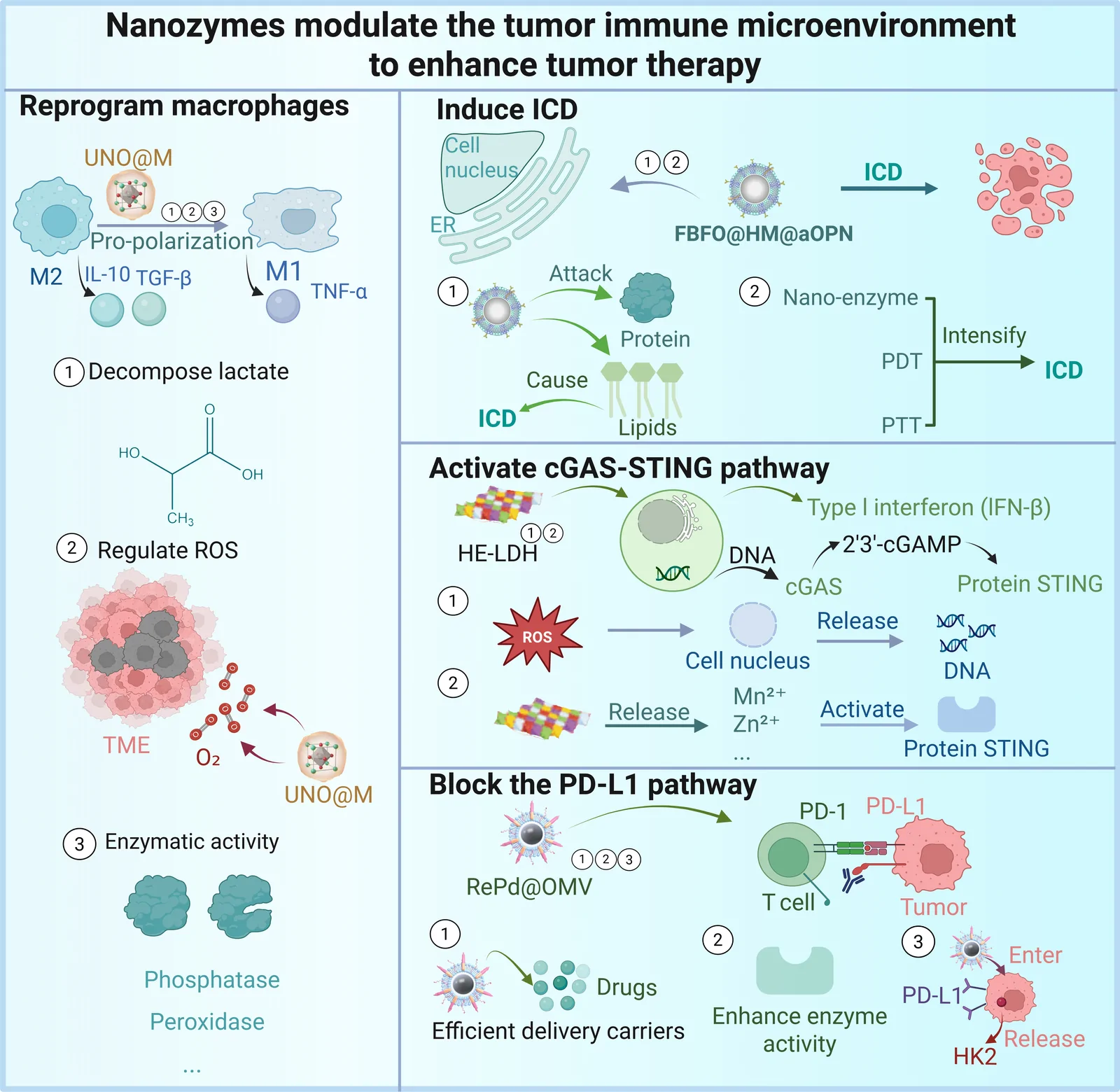
Nanozyme-mediated reprogramming of the tumor immune microenvironment. Antitumor immunity is enhanced through nanozyme-mediated regulation of immune cell function and induction of ICD. Innate immune pathways, including cGAS–STING, are also activated, while PD-1/PD-L1-targeted interventions are facilitated. Through these mechanisms, immunosuppression is alleviated and antitumor immune responses are strengthened.

### Regulation of immune cell function and phenotype

TAMs are major regulators within TIME. Although their functional states span a continuum, they are commonly grouped for operational purposes into pro-inflammatory M1-like and alternatively activated M2-like phenotypes. M1-like TAMs produce inflammatory mediators and help restrain tumor growth. M2-like TAMs secrete immunosuppressive factors and support angiogenesis, reshaping the local microenvironment in ways that favor immune escape and malignant progression. This polarization remains reversible, so TAMs can change their functional state as local cues vary. Nanozymes can influence this plasticity by degrading lactate and controlling ROS through enzyme-mimetic catalysis, promoting a shift from an M2-like state toward an M1-like phenotype [166,167].

Through their phosphatase-like activity, the lanthanum nickel oxide nanozymes reported by Baimanov et al. [168] induce macrophage autophagy, which repolarizes TAMs from an M2-like state toward an M1-like state and strengthens antitumor activity. The sulfur vacancy-rich 1T2H-MoS₂ nanozyme developed by He’s team [169] exhibits approximately 12-fold higher POD-like activity; this increase promotes TAM repolarization toward the M1 phenotype and contributes to direct tumor cell elimination. Fan et al. [170] paired a light-responsive nanozyme system with PDT, using ferroptosis to promote tumor antigen release while enabling on-demand delivery of the R848 agonist. This combination promotes the repolarization of M2 macrophages toward an M1-like phenotype and modulates immunity at multiple levels. These studies demonstrate the multiple mechanisms and application potential of nanozymes in reprogramming macrophage polarization [171–173].

In addition, nanozymes can also mimic or activate a variety of immune cells. By precisely controlling the alloy ratio, Meng and coworkers [76] developed Au_1_Pd_3_ ultra-micro alloy nanozymes that efficiently emulate the SOD-MPO cascade reaction of neutrophils, thereby triggering DNA damage and apoptosis in tumor cells. He’s team [174] reported that ECAu@CD nanoparticles (NPs) can effectively recruit and activate T cells, NK cells, and macrophages, reversing “cold” tumors into “hot” tumors and activating systemic antitumor immunity. Wang and coworkers [175] engineered CZFT (Zr/Fe-dual-doped hollow CeO₂ nanozyme combined with tirapazamine) nanoparticles to potentiate NK and CD8^+^ T cell-mediated antitumor immunity. Through the precise, spatiotemporally controllable regulation of ROS, these platforms successfully thwart both primary tumor growth and systemic metastatic spread. The above studies demonstrate the diversity of strategies for nanozymes to precisely intervene in immune cell function and synergistically reverse immunosuppression from different perspectives.

### Regulation of antitumor immune signaling

#### Induction of immunogenic cell death

Immunogenic cell death (ICD) is a specialized form of regulated cell death that links tumor cell elimination to adaptive immune activation. It is characterized by the release of DAMPs from dying cells, including calreticulin exposure, extracellular adenosine triphosphate (ATP) release, and high-mobility group box 1 (HMGB1) secretion. These immunogenic signals facilitate DC maturation, promote tumor antigen uptake, and support migration to lymph nodes, where tumor-specific CD8^+^ T cell responses and long-term immune memory can be established. Hydroxyl radicals produced by POD-like nanozymes can induce oxidative damage to endoplasmic reticulum proteins and lipids, triggering endoplasmic reticulum stress and activating ICD pathways [176]. In addition to killing tumor cells directly, nanozymes can regulate immune responses through ICD induction, thereby remodeling TIME and improving the efficacy of cancer immunotherapy [177–179].

The multifunctional ^G/AP^H-M nanoplatform reported by Xie et al. [180] induces ICD in glioblastoma through coordinated regulation of hypoxia and metabolic conditions. By modulating these tumor-associated factors simultaneously, the response to radio-immunotherapy is enhanced. Gao’s group [181] developed an injectable composite hydrogel system (MC@PHDA), in which a molybdenum disulfide (${\text{MoS}}_{2}$) nanozyme and a Chlorella-derived photosensitizer were incorporated into a hyaluronic acid-dopamine/2-aminomethacrylate matrix. Within this system, PDT and PTT are integrated to induce ICD and activate antitumor immunity.

Nanozyme-based treatment can link ICD with other cell death pathways and immunoregulatory mechanisms, allowing intervention at several biological levels. In the iron-based MOF nanozymes developed by Jiang’s team, POD/CAT-like activities are enhanced and GSH is depleted. Ferroptosis and ICD are consequently induced in parallel. These forms of cell death promote DC maturation and T cell recruitment, leading to systemic antitumor immunity [176]. The multifunctional photodynamic-enhanced biomimetic nanoplatform constructed by Zhao et al. [115] responds to the TME, induces ICD, and promotes the polarization of tumor-associated macrophages toward antitumor phenotypes. Through coordinated regulation of innate and adaptive immunity, this platform suppresses tumor progression. Collectively, these studies highlight the versatility of nanozymes in ICD-based immunotherapy and support their potential for developing combination therapeutic strategies [170,182,183].

#### Activation of the cGAS–STING pathway

The cGAS–STING pathway is a central component of innate immune signaling and functions primarily in the recognition of aberrant cytosolic DNA fragments. Activation of this pathway induces the expression of immune-related mediators, including type I interferons, and contributes to the initiation of antitumor immune responses. However, during tumor progression, many tumors acquire strategies to suppress cGAS–STING signaling, resulting in impaired immune activation and the development of “immune-silent” and “cold tumor” phenotypes.

Nanozymes can activate the cGAS–STING pathway through the combined effects of enzyme-like catalysis and physicochemical regulation. At the catalytic level, ROS generated by nanozyme-mediated reactions can induce mitochondrial and nuclear DNA damage, resulting in the release of cytosolic DNA fragments that serve as endogenous substrates for cGAS recognition [184]. Beyond conventional enzyme-mimetic functions, some metal-based nanozymes can release metal cations during TME-responsive degradation, and these ions may act as endogenous modulators or activators of cGAS–STING signaling. Existing research has shown that metal ions such as manganese (Mn^2+^) [185], zinc (Zn^2+^) [186], and cobalt (Co^2+^) [187] can directly bind to cGAS/STING proteins or increase their ligand affinity, thereby significantly enhancing downstream signal transduction [188–190].

The manganese-doped nanozyme developed by Chen et al. [185] combines Mn^2+^ release with intrinsic catalysis. Its catalytic activity generates ROS and consequent DNA damage signals, while the released Mn^2+^ modulates cGAS–STING pathway activation. This combination of catalytically induced DNA damage and ion-mediated immune regulation enhances the antitumor activity of T cells and NK cells. Similarly, the high-entropy 2D layered double hydroxide (LDH) nanoplatform developed by Wang et al. [70] drives cascade catalysis to reprogram redox homeostasis and facilitates the liquid–liquid phase separation (LLPS) of cGAS through localized Zn^2+^ release. The efficacy of innate cancer immunotherapy is thereby substantially enhanced.

Nanozymes can also be integrated with other therapeutic strategies to modulate cGAS–STING signaling. The mitochondria-targeting nano-agonist developed by Xu et al. induces oxidative stress through dual nanozyme activities and enhances cGAS–STING activation when combined with photosensitizers. Tao and coworkers [79] used hollow Prussian blue nanozymes to link ROS-mediated ICD with cGAS–STING activation. Primary tumors and metastatic lesions were suppressed more effectively. In the multimetal nanozymes developed by Qu et al. [119], CAT-like activity relieves tumor hypoxia, while photothermal effects and POD-like catalysis induce ICD. Antitumor immunity is activated through the convergence of these mechanisms.

Across these systems, cGAS–STING signaling is engaged through routes that differ mechanistically and may act in concert. This range of activity supports the use of nanozyme platforms to improve antitumor responses and may inform the design of immunotherapeutic regimens [191–193].

#### Nanozyme-enabled modulation of the PD-1/PD-L1 axis

The PD-1/PD-L1 pathway is a key immune checkpoint mechanism that maintains immune tolerance through inhibitory signals. Tumor cells highly express PD-L1, which binds to PD-1 on T cells, inhibiting T cell activation and leading to immune evasion. In addition to relieving hypoxia and indirectly down-regulating PD-L1 expression, nanozymes can serve as efficient carriers for the targeted delivery of checkpoint-targeting agents, thereby enhancing local drug accumulation, reducing systemic adverse effects, and potentiating antitumor immunity [178].

When mediating such immune checkpoint blockade therapies, it is crucial to clearly define the catalytic remodeling and carrier delivery functions of nanozymes. The intelligent biomimetic immune organelle platform developed by Wu et al. [177] not only serves as a targeted carrier to deliver and release PD-L1 nanobodies to physically block inhibitory signals, but its core rhenium–palladium (Re–Pd) bimetallic nanozyme component also plays a key role in biochemical remodeling. This nanozyme component induces strong oxidative stress and ICD locally through significant POD and GSH-OXD activities, thereby promoting the recruitment and activation of T cells. This nanozyme-driven priming of the TIME establishes a favorable immunological landscape for PD-L1 nanobodies to reverse T cell exhaustion, thereby achieving a potent therapeutic synergy between in situ catalytic immune activation and antibody-targeted blockade.

Zhu’s team combined single-atom nanozymes with immune checkpoint blockade, which enhanced the efficacy of anti-PD-1 antibodies by inducing ICD, relieving hypoxia, and regulating chemokines [194]. The gadolinium–palladium nanoplatform constructed by Sun’s group enhances nanozyme activity through GOx cascade, regulates the AMPK/CMTM6 pathway to inhibit PD-L1 expression, and overcomes radiotherapy-induced immunosuppression [121].

Recent studies have revealed the link between copper metabolism and PD-L1 regulation. The copper-based hybrid nanozyme developed by Cai et al. [195] enhances bladder cancer immunotherapy by activating AMPK to induce ferrophagy, up-regulating p53, and reducing PD-L1 levels. The Pt@PCN-Cu nanozyme synthesized by Wang et al. [149] induces cuproptosis and regulates PD-L1 through HK2, significantly enhancing the efficacy of anti-PD-L1 combined therapy for pancreatic cancer. These studies demonstrate that nanozyme-based systems can enhance PD-1/PD-L1-targeted immunotherapy through multiple mechanisms, including targeted delivery of checkpoint inhibitors, indirect down-regulation of PD-L1 expression, and sensitization to immune checkpoint blockade.

Through precise catalysis, nanozymes can reprogram macrophages and induce ICD, while activation of cGAS–STING further contributes to the reversal of local immunosuppression [196,197]. Long-term immune remodeling is constrained by complex regulatory mechanisms within TIME. At the cellular level, the paracrine immunosuppressive network formed among TIME components can promote adaptive resistance after immune stimulation, causing activated M1 macrophages to acquire a tumor-supportive phenotype [198]. At the pathway level, responses to ICD induction vary considerably across molecular subtypes. cGAS–STING activation is also governed by the availability of cytosolic DNA substrates and several regulatory factors. These include epigenetic suppression of endogenous STING expression and DNA degradation mediated by intracellular exonucleases. In the future, engineering nanoassemblies with multi-input logic-gated responsiveness to execute programmed, orthogonal interventions across target cells and immunosuppressive pathways, alongside deciphering the dynamic nonlinear crosstalk between nanocatalysis and adaptive immune networks, will be paramount to surmounting the current mechanistic bottlenecks in this field.

In summary, tumor redox homeostasis, metabolic processes, and the immune microenvironment are interconnected, forming a highly intertwined biochemical and immune regulatory network in tumor evolution. Nanozymes, by targeting multiple sites within this network, achieve a cascade of interventions ranging from correction of biochemical imbalances to antitumor immune remodeling. However, this network’s efficacy is constrained by biophysical and microecological barriers: Abnormal tumor vessels impede transport, while the intratumoral microbiota shapes immune surveillance and drug resistance. Therefore, extending the regulation strategy of nanozymes to tumor blood vessels and microbiota is a key entry point for achieving multidimensional and cross-scale synergistic remodeling of the TME.

## Regulation of Additional Tumor Microenvironmental Determinants by Nanozymes

### Modulation of the abnormal tumor vascular microenvironment

Abnormal vascular networks in tumors are key physical and physiological barriers driving malignant tumor progression and treatment resistance. Compared with normal vasculature, tumor blood vessels are characterized by structural abnormalities, including irregular organization, incomplete vessel walls, reduced pericyte coverage, and disrupted basement membranes. These defects result in increased vascular permeability and insufficient blood perfusion [11,199]. Such vascular dysfunction further aggravates intratumoral hypoxia, extracellular acidification, and immunosuppressive conditions while limiting the delivery of therapeutic agents and immune cell infiltration. To overcome these limitations, nanozyme-based vascular regulation strategies have mainly been developed through 3 approaches: vascular ablation, anti-angiogenesis, and vascular normalization [200,201].

Conventional vascular ablation and anti-angiogenic strategies aim to restrict tumor growth by disrupting the tumor blood supply. During vascular ablation, nanozyme-generated ROS directly damage endothelial cells. Anti-angiogenic treatment relies on inhibitor payloads that interrupt vascular endothelial growth factor (VEGF) signaling and reduce new vessel formation. Both approaches can intensify intratumoral hypoxia, restricting the penetration of chemotherapeutic agents and the recruitment of effector immune cells [202]. Vascular normalization aims for the temporary recovery of vessel structure and function. Through nitric oxide synthase (NOS)- and CAT-like activities [203], endothelial barrier function is improved and pericyte coverage is increased, supporting vascular recovery [204]. Excessive interstitial fluid pressure (IFP) is reduced, and blood perfusion improves, which limits the extent of hypoxic regions. Antitumor drugs are consequently delivered more efficiently, and effector immune cells, including cytotoxic T lymphocytes, can enter tumor tissue more readily [205].

These mechanisms have been incorporated into several nanozyme platforms. Zhao et al. [115] developed FBFO@HM@aOPN as a biomimetic platform with stimulus-responsive behavior. Oxygen is generated catalytically, relieving intratumoral hypoxia and promoting vascular remodeling; the accumulation and penetration of therapeutic agents in tumor tissue are improved as a result. Wang et al. [206] designed the HRRu (a coassembled heme, Fmoc-protected arginine, and Ru^3+^ nanozyme) nanozyme to regulate gaseous signaling through its NOS- and CAT-like activities. A heme cofactor, Fmoc-protected arginine, and Ru^3+^ ions are integrated into the system, enabling NO and O_2_ to be released autonomously through an H_2_O_2_-triggered cascade. Their simultaneous release promotes vascular normalization and substantially improves blood perfusion. By relieving intratumoral hypoxia, this process also reduces a source of therapeutic resistance.

In anti-angiogenic strategies, nanozymes offer a distinct combination of carrier function and biochemical regulation. Demissie and coworkers [145] engineered HPDA@TPZ/${\text{MoS}}_{2}$NBs by integrating ${\text{MoS}}_{2}$ nanozymes with the hypoxia-activated prodrug tirapazamine (TPZ). This system effectively blocks the migration and proliferation of endothelial cells by specifically binding to VEGF-A_165_, exhibiting multi-level targeted anti-angiogenic properties. Unlike angiogenesis normalization, this system cleverly utilizes the extreme hypoxic microenvironment inevitably caused by anti-angiogenesis to precisely activate the cytotoxicity of TPZ, providing a new paradigm for the synergistic regulation of anti-angiogenesis and hypoxia-sensitized killing.

### Modulation of the tumor-associated microbial microenvironment

The tumor microbiota, as an emerging and indispensable component of the TME, plays a crucial regulatory role in tumorigenesis, metastasis, immune monitoring, and prognosis.

Accumulating evidence demonstrates that aberrant intratumoral colonization by specific pathogens substantially heightens therapeutic resistance and fuels distant metastasis through multifaceted mechanisms. These include the direct enzymatic inactivation of chemotherapeutic agents, activation of anti-apoptotic cascades and cytoprotective autophagy, attenuation of radiotherapeutic and ROS-driven cytotoxicity, and orchestration of an immunosuppressive microenvironment [207–210].

Existing research has confirmed that certain nanozymes can have both antibacterial and anticancer functions, such as praseodymium-doped CoFe LDHs and cobalt(II)-yttrium(III)-iron(III) LDHs [211,212]. Recent research has focused on restoring tumor treatment sensitivity by precisely regulating or specifically eliminating tumor-colonizing bacteria to break the mutually beneficial “pathogen–tumor symbiosis” of pathogen-driven drug resistance barriers. On the one hand, phage-guided nanosystems and biomimetic nanodelivery platforms camouflaged with tumor or bacterial cell membranes selectively eradicate intratumoral *Fusobacterium nucleatum*, thereby directly reversing pathogen-mediated immunosuppression and chemoresistance while suppressing metastatic dissemination [207–209]. On the other hand, converging targeted antibacterial therapy with catalytic nanomedicine represents a cutting-edge paradigm for TME reprogramming. For instance, the protein-supported copper single-atom nanozyme (BSA-Cu SAN) engineered by Wang et al. [213] efficiently catalyzes ROS generation and GSH depletion in response to tumor microenvironmental cues. This dual catalytic activity enables in situ elimination of intratumoral *F. nucleatum* while disrupting pathogen-associated anti-apoptotic signaling and cytoprotective autophagy. Nanozyme catalysis also increases oxidative stress within tumors, causing extensive apoptosis and strengthening antitumor responses.

The structural adaptability of nanozymes and their capacity for targeted delivery could be used to build systems that regulate tumor-associated microbiota and improve treatment outcomes, an approach with potential for future cancer therapy (Fig. S2).

## Summary and Outlook

Work on nanozyme regulation of the TME now occupies a substantial place in cancer nanomedicine [30]. This review considers the physicochemical properties of nanomaterials within the framework of catalytic medicine, asking how nanozymes engage pathological conditions in the TME at the molecular level. Endogenous substrates present at tumor sites can be consumed in situ by materials with enzyme-like activity. Through these reactions, redox homeostasis is disturbed and metabolic programs are altered; effects on the immune microenvironment may follow, allowing remodeling across these biological levels to be coordinated [26,214,215]. Destabilizing the conditions that sustain tumor survival offers a mechanistic means of addressing resistance to established therapies [25]; flexibility in material design and the capacity for functional integration also enable biological pathways to be regulated together [216]. Natural enzymes generally catalyze a defined reaction, while a nanozyme can be structurally engineered to contain several enzyme-like activities. Multiple targets in the pathological microenvironment may thus be engaged by the same platform [217–219]. Nanozymes constructed from inorganic materials or MOFs also tend to retain physicochemical stability under demanding conditions. Nanozyme catalysis can be tuned at the design stage, and many of these materials are amenable to large-scale production. These features support the construction of platforms that combine diagnostic and therapeutic functions [31,34,43–48] (Fig. 5).

**Fig. 5.**
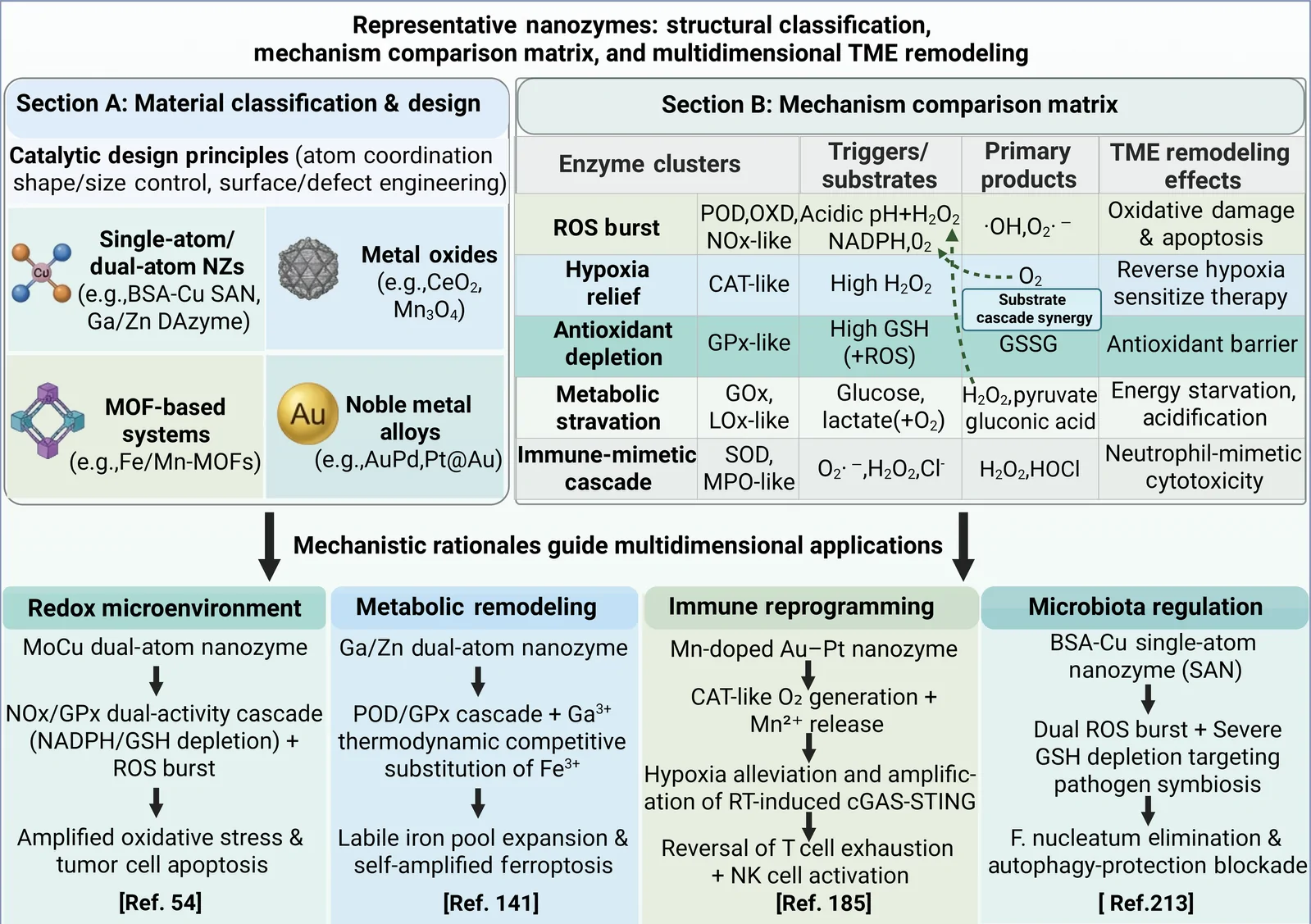
Structural classification and catalytic mechanisms of representative nanozymes for TME remodeling. Major material classes, including single-atom and diatomic nanozymes, metal oxides, MOF-based systems, and noble-metal alloys, are summarized together with their catalytic design principles, substrates and products, and effects on redox homeostasis, metabolic reprogramming, immune regulation, and tumor-associated microbiota.

Preclinical proof-of-concept work has produced encouraging results. Claims of clinical readiness, however, have advanced faster than the pharmacokinetic and translational evidence needed to support them. Targeted delivery must be evaluated alongside biodistribution and systemic safety. Accumulation at tumor sites is typically modest because circulating particles are rapidly sequestered by the reticuloendothelial system (RES). Following intravenous administration, a protein corona forms as serum proteins adsorb onto the nanozyme surface. Once formed, the corona can cover catalytic sites and promote off-target toxicity, decreasing catalytic efficiency in vivo [31]. A substantial share of the administered material is subsequently retained in the liver and spleen, reducing the therapeutic dose that reaches deep tumor tissue. Persistent accumulation also raises concern about chronic toxicity in major organs, including the heart, liver, spleen, lungs, and kidneys. The rigidity of lattices containing transition or heavy metals helps preserve catalytic durability, although the same property may prolong tissue residence [41,111]. Mechanistic questions remain. Therapeutic phenotypes are commonly documented after nanozyme administration; direct examination of transport across physical barriers in the TME and activation of specific intracellular pathways is still required. Overengineering, particularly arbitrary component integration, introduces a related concern. In some studies, established combination schemes are expanded by adding several elements before their structure–property relationships have been characterized in sufficient depth. Where biochemical synergy is modest, the additional components increase biological complexity and make material-related risks harder to manage.

Clinical translation therefore requires a rigorous appraisal of the therapeutic niche of nanozymes relative to current standards of care. Most studies rely heavily on subcutaneous xenograft models of a single tumor type, and parallel validation across cancer types remains limited. Consequently, the ability of one nanozyme design to address the marked heterogeneity among tumor types remains uncertain. Within precision oncology, nanozymes generally provide lower molecular specificity than targeted biologics, and their efficacy depends strongly on the physicochemical conditions of the TME. Their most realistic translational role is as microenvironment-priming agents or sensitizing co-therapies used alongside targeted treatments. Within this role, catalytic activity is used to alter the local reaction environment, while molecular recognition is provided by the accompanying targeted agent. By disrupting biophysical and biochemical barriers through catalytic remodeling, nanozymes may restore the activity of targeted modalities weakened by intratumoral heterogeneity.

Progress from bench to bedside will require the field to shift its emphasis from adding material components to addressing defined clinical needs with designs that are rational and standardized. Research on new materials in this field should begin with the principles of nanocatalysis. In the design of single-atom catalysts (SACs), artificial intelligence can be combined with density functional theory (DFT) to refine catalytic activity and selectivity at the atomic scale. Behavior at biological interfaces must be considered together with systemic clearance. One possible design pairs an antifouling surface with a degradable polymer that responds to the microenvironment. Hepatic retention could then be reduced while catalytic robustness is maintained, improving the balance between clearance and therapeutic efficacy. Progress toward the clinic also depends on a shared basis for evaluation. Studies vary in material preparation and in the methods used to quantify active units; dosing practices differ as well. Stringent chemistry, manufacturing, and controls (CMC) guidelines based on standards for natural enzymes would allow materials produced in different laboratories to be compared meaningfully. Benchmarking under systematically controlled conditions is needed to determine the biological efficacy attributable to each structural design. Preclinical testing should also be broadened to include 3D tumor organoids and humanized immune mouse models. Nanocatalytic signal-transduction networks in complex TMEs can then be mapped at high resolution using single-cell multi-omics and spatial transcriptomics, helping identify therapeutic targets. Rigorous investigation of mechanism, accompanied by continued refinement of concepts, will determine how nanozyme platforms with tunable properties can be incorporated into personalized combination therapy.

Research on nanozyme-mediated TME modulation is moving from early proof-of-concept studies toward development focused on clinical translation. Continued integration of materials science, tumor biology, nanomedicine, and catalytic therapy may establish nanozymes as a useful platform in precision oncology, with the potential to address therapeutic resistance and improve clinical outcomes (Fig. S3).
