# Supplementary material for: Nanozymes in Tumor Microenvironment Modulation for Cancer Therapy
Source: Biomater Res. 2026 Oct 7;30:0425. doi: 10.34133/bmr.0425 (PMC13643025; doi:10.34133/bmr.0425)
Supplement: Supplementary file 1 [file bmr.0425.f1.docx]

**Supplementary Figure S1. Major enzyme-like activities of nanozymes for cancer therapy**. Representative POD-, OXD-, CAT-, GOx-, NOX-, LOX-, GPx-, SOD-, and MPO-like activities of nanozymes and their corresponding catalytic mechanisms are illustrated.

**Supplementary Figure S2.** **Regulation of additional tumor microenvironmental determinants by nanozymes.** Beyond redox, metabolic, and immune regulation, nanozymes can also modulate abnormal vascular and microbial microenvironments. These effects may improve tumor perfusion, reduce microenvironment-associated resistance, and further enhance therapeutic efficacy.

**Supplementary Figure S3. Advantages, challenges, and future directions of nanozymes for tumor microenvironment modulation**. This figure summarizes the major strengths, translational barriers, and future development priorities of nanozymes in cancer therapy.
